# Step-Double-Zone-JTE for SiC Devices with Increased Tolerance to JTE Dose and Surface Charges

**DOI:** 10.3390/mi9120610

**Published:** 2018-11-22

**Authors:** Yifei Huang, Ying Wang, Xiaofei Kuang, Wenju Wang, Jianxiang Tang, Youlei Sun

**Affiliations:** Key Laboratory of RF Circuits and Systems, Ministry of Education, Hangzhou Dianzi University, Hangzhou 310018, China; 162040160@hdu.edu.cn (Y.H.); kuangxiaofei@hdu.edu.cn (X.K.); 162040172@hdu.edu.cn (W.W.); hztangjx@hdu.edu.cn (J.T.); 161040013@hdu.edu.cn (Y.S.)

**Keywords:** edge termination, silicon carbide (SiC), junction termination extension (JTE), breakdown voltage (BV)

## Abstract

In this paper, an edge termination structure, referred to as step-double-zone junction termination extension (Step-DZ-JTE), is proposed. Step-DZ-JTE further improves the distribution of the electric field (EF) by its own step shape. Step-DZ-JTE and other termination structures are investigated for comparison using numerical simulations. Step-DZ-JTE greatly reduces the sensitivity of breakdown voltage (BV) and surface charges (SC). For a 30-μm thick epi-layer, the optimized Step-DZ-JTE shows 90% of the theoretical BV with a wide tolerance of 12.2 × 10^12^ cm^−2^ to the JTE dose and 85% of the theoretical BV with an improved tolerance of 3.7 × 10^12^ cm^−2^ to the positive SC are obtained. Furthermore, when combined with the field plate technique, the performance of the Step-DZ-JTE is further improved.

## 1. Introduction

Silicon carbide (SiC), a representative of the third generation of semiconductor materials, is a promising candidate for power devices due to its superior properties such as wide bandgap, high breakdown electric field, high thermal conductivity, and high drift saturation speed [1,2,3,4,5]. Hence, SiC devices are more suitable than silicon counterparts for high-voltage, high-frequency, and high-temperature applications. However, the potential performance (e.g., high blocking) of SiC materials is limited due to the presence of the effect of field crowding at the device edge.

To achieve high breakdown voltages (BV) for the planar junction close to its theoretical voltage, using a proper edge termination structures is essential. In the past few decades, a large number of edge termination structures have been investigated and applied in SiC power devices, including field plate (FP) [6,7], floating field rings (FFRs) [8,9,10], and junction termination extension (JTE) [11,12,13]. FP is easy to design and fabricate, but introduces electric field (EF) peaks at the end, which limits their application to high-voltage devices. FFRs are widely used in power devices because they can be formed at the same time as the P+ region in a PIN or junction barrier Schottky diode, while this reduces the number of processing steps, but FFRs are more demanding to design with specific ring requirements such as width, spacing, and number of rings. Among them, JTE is a prevalent and highly efficient edge termination structure for SiC power devices. The effective JTE can sustain high BV, but is strongly dependent on precise JTE dose control, which is a big challenge. The BV for conventional single-zone junction termination extension (SZ-JTE) is very sensitive to JTE dose. Feng [14] investigated PIN diodes in 4H-SiC with different terminal structures. The results showed that, for 90% of the theoretical BV, a conventional SZ-JTE obtained a narrow JTE dose tolerance of 1.0 × 10^12^ cm^−2^. Therefore, many modified forms of JTE have been proposed to improve the sensitivity, such as guard ring-assisted JTE (GA-JTE) [15,16,17], double-zone JTE (DZ-JTE) [18,19], multiple-zone JTE (MZ-JTE) [20,21], etched JTE [22,23], counter-doped JTE (CD-JTE) [24], and mesa combined with JTEs [25,26]. Feng [14] also reported that the JTE dose tolerance (4.8 × 10^12^ cm^−2^) in the conventional DZ-JTE was improved compared with the conventional SZ-JTE. Huang [24] proposed and investigated PIN diodes with CD-JTE and other conventional terminal structures. The simulation results in his paper showed the CD-JTE with a JTE dose tolerance of 11.0 × 10^12^ cm^−2^ was greatly improved compared with other structures.

In this paper, an edge termination structure called Step-DZ-JTE for 4H-SiC PIN diode is proposed and investigated. Combined with conventional DZ-JTE, the proposed structure changes the shape of DZ-JTE to a step type to adjust the distribution of the electric field. On the one hand, the Step-DZ-JTE does not add to the number of p-type implants. On the other hand, the simulation results show that a wide tolerance of 12.2 × 10^12^ cm^−2^ is obtained for the Step-DZ-JTE, which is greatly improved over the conventional DZ-JTE and slightly improved compared with the CD-JTE. Moreover, when combined with the FP technology, the performance of the Step-DZ-JTE is further improved, as verified by numerical simulations.

## 2. Materials and Methods

### 2.1. Device Structure

**Figure 1:**
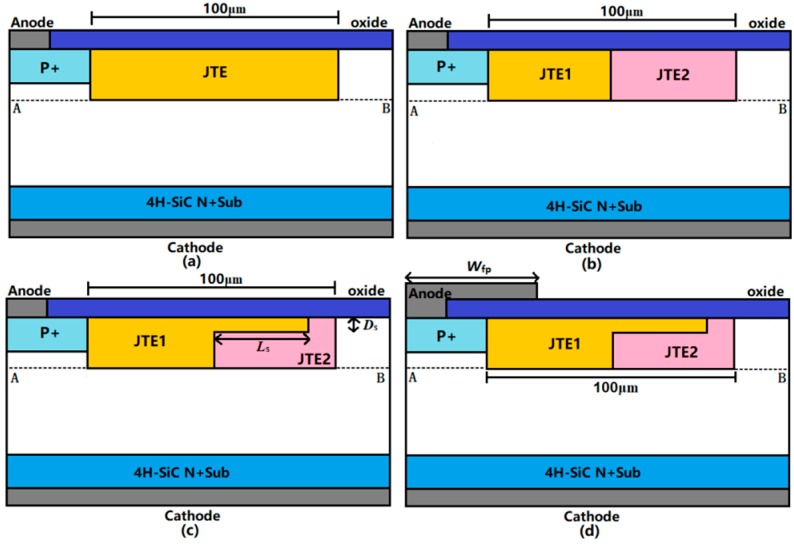
Schematic structures of (**a**) single-zone junction termination extension (SZ-JTE); (**b**) double-zone JTE (DZ-JTE); (**c**) Step-DZ-JTE; (**d**) Step-DZ-JTE with field plate (FP). The n- epi-layer is 30-µm thick and 2 × 10^15^ cm^−3^ doped.

In this section, a 4H-SiC PIN diode with a 30-μm epi-layer doped at 3 × 10^15^ cm^−3^ can attain 4000 V from the ideal parallel junction [27]. All termination structures for PIN diodes were investigated using Silvaco TCAD 2-D device simulations (atlas 5.22.1.R, Silvaco Inc., Santa Clara, CA, USA). The breakdown condition was defined as the point at which the calculated impact ionization integral reaches unity [23]. The major models used in simulations include Schokley‒Read‒Hall (SRH), Auger recombination, impact ionization, and incomplete ionization. Cross-sectional views of PIN diodes with three termination structures are shown in Figure 1, including: (a) single-zone JTE (SZ-JTE); (b) double-zone JTE (DZ-JTE); (c) Step-DZ-JTE; (d) Step-DZ-JTE with FP. The feature of Step-DZ-JTE is a step structure, which improves EF distribution by adjusting the depth and length of the step JTE. In addition, Step-DZ-JTE with FP can reduce the amount of EF crowding near the main junction in a low JTE dose by adding an anode FP. In this figure “*D*_s_”, “*L*_s_”, “*W*_fp_” are the depth and length of step JTE1, and the length of the anode FP, respectively. Among these structures, the length of termination region is fixed at 100 µm, where the simulated BV of the JTE starts to saturate. The values of the major optimized parameters of the proposed structure are summarized in Table 1.

### 2.2. Fabrication Procedure

**Figure 2:**
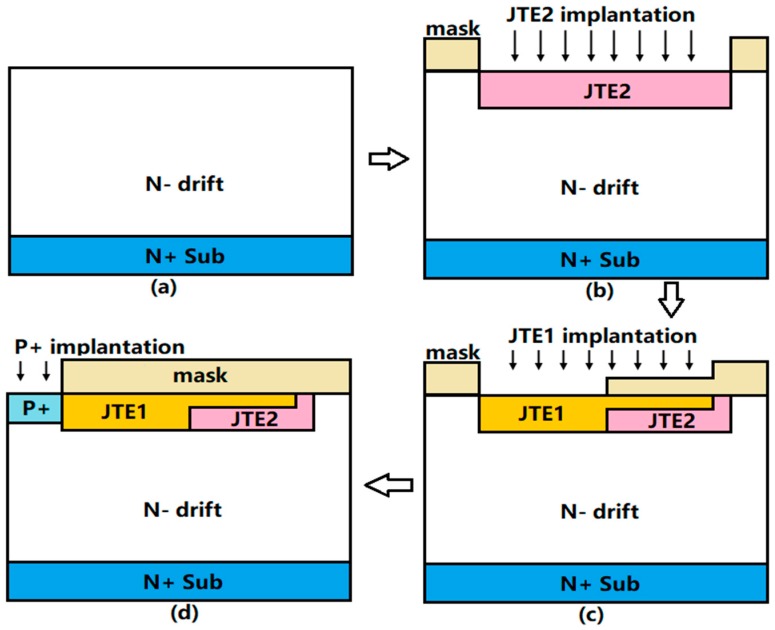
Fabrication procedure of making step JTE in the Step-DZ-JTE. (**a**) Base layers. (**b**) JTE2 region formed by ion implantation. (**c**) JTE1 region fromed by ion implantation with the specific mask. (**d**) Main junction P+ formation.

Figure 2 shows a feasible fabrication procedure for building a step JTE in the Step-DZ-JTE. As shown in Figure 2a, an n- 4H-SiC epitaxial layer is first grown on an n+ 4H-SiC substrate. Then, SiO_2_ layer as the mask materials are thermally grown on the epitaxial layer and a photoresist is patterned on the mask to form a JTE2 window [28]. Next, multiple aluminum implantations are applied to form the JTE2 region. Then, a graphite cap layer is grown on the surface after removing the mask material to prevent the sublimation and roughening of the surface during the next annealing [28,29]. Next the implantations of the JTE2 are activated by high temperature annealing to activate acceptor impurities and form a box profile, as shown in Figure 2b. In order to form the step distribution of the JTE1, the step mask, as shown in Figure 2c is the key to the formation of the JTE and its shape can be form by the etching process. Similarly, the JTE1 region is formed using ion implantation through the mask and then activation annealing. Finally, the main P+ region is formed using ion implantation with high doses, as shown in Figure 2d. All annealing conditions are implemented at the temperature of 1650 °C under argon ambient with the graphite cap [30]. This process is easier to implement than etching, and it avoids the extra interface charges caused by filling the dielectric after etching.

## 3. Results

### 3.1. Simulation Optimization of the DZ-JTE

**Figure 3:**
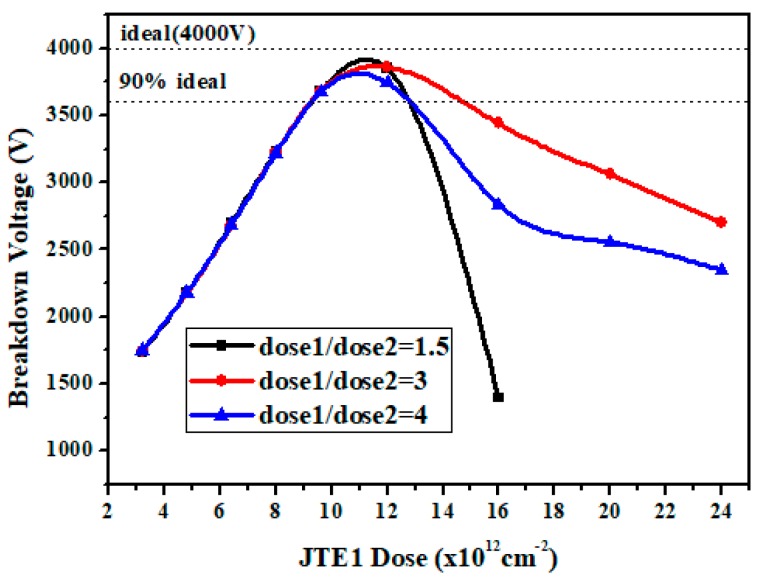
Simulated breakdown voltage (BV) versus ratio of the doses with different doses for the DZ-JTE optimization.

Based on the optimized conventional SZ-JTE, this section discusses the optimization of the DZ-JTE, which mainly involves the length and dose of the double JTE. Figure 3 shows the simulated BV versus the ratio of dose1 in JTE1 to dose2 in JTE2 for DZ-JTE. In the higher JTE1 dose range, the ratio of doses has a significant effect on the BV. The BV increases as the ratio increases, and then drops sharply. There is a reasonable ratio value of doses to avoid a sharp decrease in the BV. As can be seen from the figure, the optimized ratio value of dose1/dose2 is 3. 

**Figure 4:**
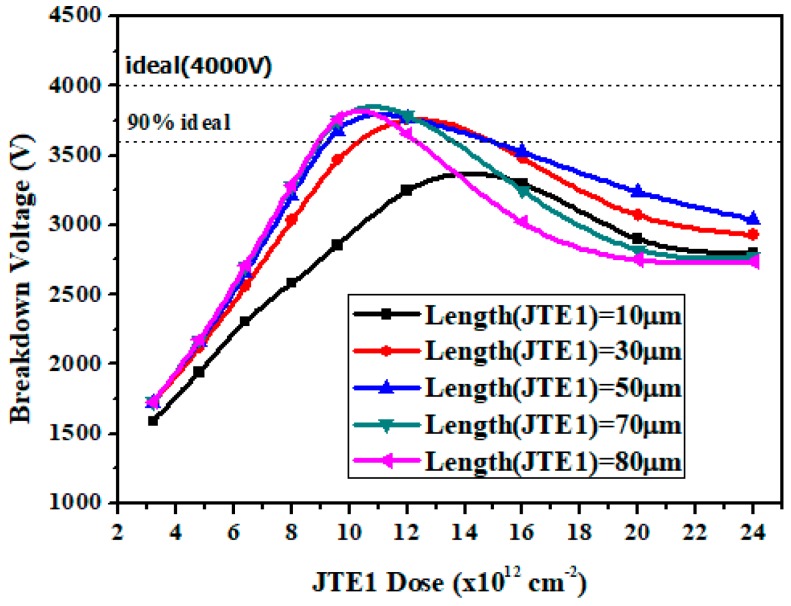
Simulated BV versus JTE1 length with different doses for the DZ-JTE optimization.

Based on the optimized ratio above, the simulated BV versus JTE1 length for DZ-JTE with different JTE1 dose is shown in Figure 4. We see that JTE1 length has little effect on BV in the lower of JTE1 dose range. This phenomenon is explained by the fact that too low a concentration makes no difference between JTE1 and JTE2. In other JTE1 doses, as JTE1 length increases, the curve gradually rises. When JTE1 length is 50 μm, the curve reaches its highest point. In addition, the curve drops rapidly as JTE1 length exceed 50 μm. This is because, when the JTE1 length is too long, the JTE2 length is shorter and the effect of JTE2 (alleviating the EF of JTE1) is reduced. 

### 3.2. Simulation Optimization of the Step-DZ-JTE with FP

**Figure 5:**
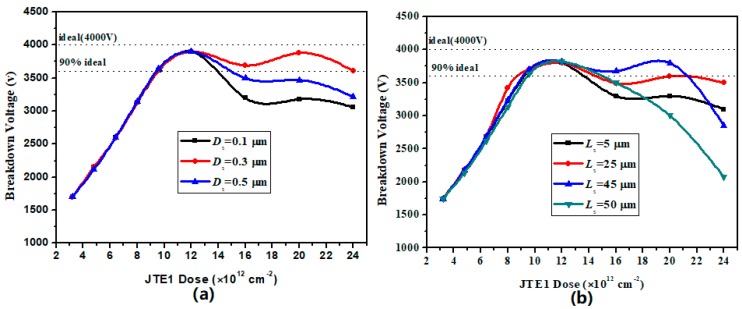
Simulated BV versus (**a**) the depth (*D*_s_) and (**b**) the length (*L*_s_) of the Step JTE for Step-DZ-JTE optimization.

Based on the optimized DZ-JTE, this section optimizes the length and depth of the step JTE1 for the proposed Step-DZ-JTE. Figure 5 shows the simulated BV versus the depth (*D*_s_) and the length (*L*_s_) of step JTE1 for Step-DZ-JTE with FP. As can be seen from Figure 5a, the curve of the BV versus JTE1 dose rises first and then declines as the *D*_s_ increases in higher of JTE1 dose range (i.e., >12 × 10^12^ cm^−2^). This is because the EF is more concentrated at the end of the step JTE1 with the *D*_s_ increases, leading to premature breakdown. When the *D*_s_ is 0.3 μm, the Step-DZ-JTE with FP attains the maximum value of the BV, meaning the optimized value of *D*_s_ is 0.3 μm. Figure 5b shows the simulated BV versus the length (*L*_s_) of step JTE1 for Step-DZ-JTE. When JTE1 dose is lower than 12 × 10^12^ cm^−2^, the *L*_s_ has no effect on the relationship between the BV and JTE dose. In the other range of JTE1 dose, the value of 45 μm is a critical value of the *L*_s_. Regardless of whether the *L*_s_ is larger or small than the value, the curves of the BV versus JTE1 dose are lower than the curve corresponding to 45 μm. It can be seen from the figure that the longer the *L*_s_ is, the faster the curves fall. This phenomenon can be explained by the fact that the longer the *L*_s_ is, the higher the carrier concentration is in the step JTE1, resulting in EF crowding at the end of the step JTE1 in the reverse blocking state.

**Figure 6:**
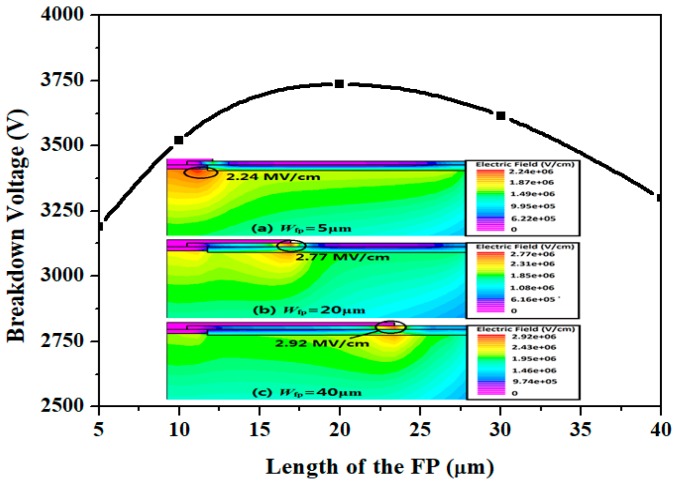
Simulated BV versus FP length (*W*_fp_) for the Step-DZ-JTE with FP optimization.

Figure 6 shows the effect of FP on the simulated BV for the Step-DZ-JTE with FP. The maximum BV is obtained when the FP length (*W*_fp_) is 20 μm. The insets of Figure 6 show simulated EF distribution at breakdown with different *W*_fp_. As shown in inset (a), the peak EF occurred near the main junction when the *W*_fp_ is less than 20 μm (e.g., 5 μm). As the *W*_fp_ increases, the EF crowding near the main can be effectively suppressed by the FP as shown in inset (b). The oxide field is 2.77 MV/cm, shown in inset (b), which is less than the oxide critical field (6 MV/cm in [31]). This means that there is no oxide degradation at breakdown. When the *W*_fp_ is more than the optimal length, the location of the peak EF shifts into the periphery of the FP, as shown in inset (c). As can be seen from inset (c), the oxide field (2.92 MV/cm) is also less than 6 MV/cm. 

### 3.3. Compare Electric Field Distribution

**Figure 7:**
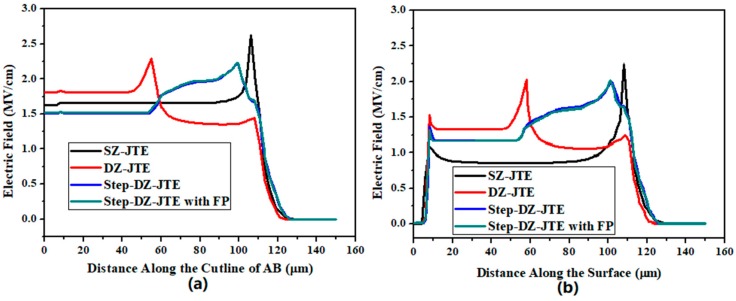
Simulated electric field distribution at OFF-state breakdown (**a**) along the cutline of AB and (**b**) on the surface for SZ-JTE, DZ-JTE, Step-DZ-JTE with FP (dose1/dose2 = 3, and JTE1 dose = 2 × 10^13^ cm^−2^).

The distribution of the electric field under the reverse blocking characteristic can reflect the problem of the conventional terminal structures and the proposed Step-DZ-JTE can be clearly compare with them. Figure 7 compares the simulated EF distribution along the cutline of AB (shown in Figure 1) and on surface of the JTE for SZ-JTE, DZ-JTE, and Step-DZ-JTE with FP at the reverse blocking voltage of 3500 V when JTE1 dose is 2 × 10^13^ cm^−2^. Regardless of the distribution of the EF along the bottom of the JTE (Figure 7a) or the distribution of the EF along the JTE surface (Figure 7b), the trend of their curves is generally the same. At this high JTE dose, the peak EF occurred at the end of JTE for the SZ-JTE, resulting in breakdown prematurely. The DZ-JTE can improve the EF at the terminal edge, but a new peak EF appears at the end of the JTE1. Under the effect of step JTE, the Step-DZ-JTE with FP further suppresses the EF crowding at the end of the JTE1. However, the effect of the FP for the Step-DZ-JTE with FP is very small. This is because that concentration of the acceptor carrier is too large at this high JTE dose, so that the effect of the FP absorbing part of the EF is not obvious.

## 4. Discussion

**Figure 8:**
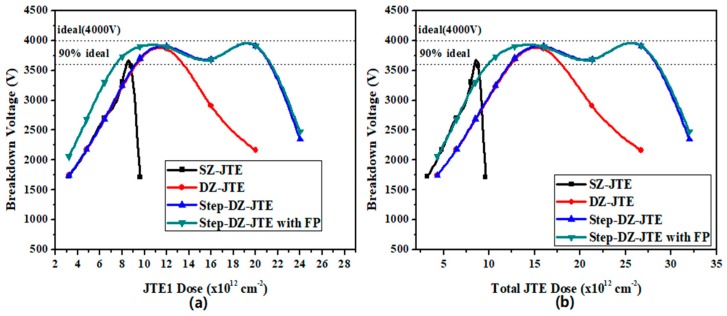
Simulated BV versus (**a**) JTE1 dose and (**b**) total JTE dose for SZ-JTE, DZ-JTE, Step-DZ-JTE without FP, and Step-DZ-JTE with FP.

In order to evaluate the performance of the Step-DZ-JTE, it is compared with other termination structures while examining the effects of JTE1 dose and surface charges (SC). First, we discuss the effect JTE dose has on the simulated BV. Figure 8 shows the BV as a function of the JTE1 dose and total JTE dose. As can be seen from Figure 8a, the SZ-JTE shows a very narrow JTE dose tolerance of 0.4 × 10^12^ cm^−2^ at 90% of the ideal BV. For the SZ-JTE, the percentage of positive and negative variation allowed to deviate from the optimized dose are +2.2% and −2.2% (positive means tolerance to allow for exceeding the optimized JTE dose; negative means tolerance to allow for less than the optimized JTE dose). By adopting two zones with different doses, the DZ-JTE shows a significant improvement of 4.1 × 10^12^ cm^−2^ at 90% of the ideal BV, of which the percentage of positive and negative variation are +17.5% and −17.0% The Step-DZ-JTE, proposed in this paper, is superior to the two termination structures mentioned above. The Step-DZ-JTE has a BV with reduced sensitivity to JTE1 dose and exhibits a wider JTE1 dose tolerance of 12.2 × 10^12^ cm^−2^ at 90% of the ideal BV of which the percentage variations are +75% and +18.4%. In the Step-DZ-JTE with FP, the curve coincides with the Step-DZ-JTE at the higher JTE dose. However, at a low JTE1 dose, the Step-DZ-JTE with FP performs better than the Step-DZ-JTE. This is because FP can relieve the EF in the main junction at low JTE1 dose. Thus, the Step-DZ-JTE creates a wide range of JTE1 dose at 90% of the ideal BV with 13.8 × 10^12^ cm^−2^, of which the percentage variations are +75% and −35%. In addition, the total JTE dose can be calculated based on the previously optimized ratio value of dose1/dose2. The relationship between the BV and total dose is shown in Figure 8b. Total JTE dose tolerance for 90% of the ideal BV with four terminal structures are listed in Table 2.

**Figure 9:**
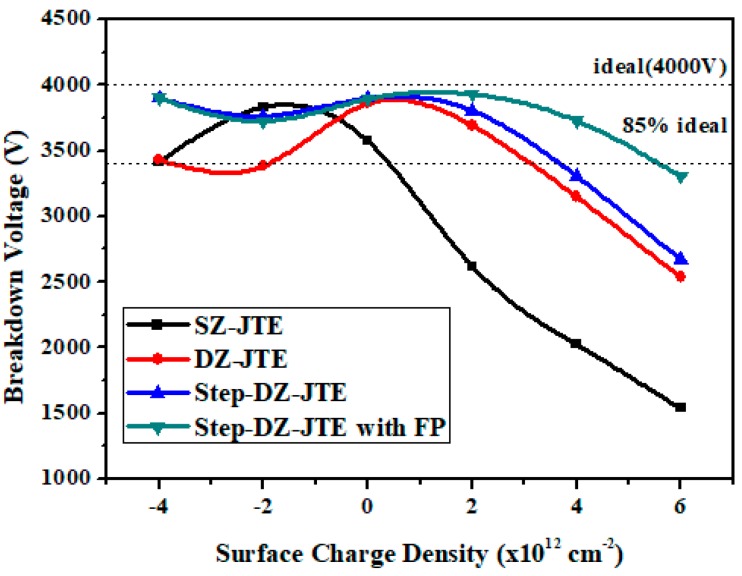
Simulated BV versus surface charges for SZ-JTE, DZ-JTE, Step-DZ-JTE without FP, and Step-DZ-JTE with FP.

On the other hand, the effect of SC on the BV is investigated since SC will affect the charge distribution and, thus, the EF distribution. In particular, positive SC has a large influence because the positive charges cancel the depleted acceptors in the JTE region. As a result, as shown in Figure 9, the SZ-JTE exhibits a very small tolerance to positive SC and obtains a positive charge density of 0.5 × 10^12^ cm^−2^ at 85% of the ideal BV. The DZ-JTE and Step-DZ-JTE show a BV with reduced sensitivity to SC and they obtain a positive charge density of 3.2 × 10^12^ cm^−2^ and 3.7 × 10^12^ cm^−2^ at 85% of the ideal BV, respectively. However, after adding the FP, the Step-DZ-JTE with FP shows the widest positive charge density of 5.5 × 10^12^ cm^−2^. Table 2 summarizes the basic performance of the four different termination structures. Compared with DZ-JTE, the number of *p*-type ion implantations for the proposed Step-DZ-JTE did not increase, and the performance of the Step-DZ-JTE is greatly improved. Combined with FP technology, the Step-DZ-JTE with FP further reduces the sensitivity of BV to JTE doses and surface charges.

## 5. Conclusions

A Step-DZ-JTE edge termination scheme is proposed in this paper and the device simulation results show that it has superior terminal performance. A comparison of the simulation results for the SZ-JTE and DZ-JTE shows that the Step-DZ-JTE greatly reduces the sensitivity of the JTE dose and SC. For a 30-µm epi-layer, the Step-DZ-JTE can exhibits a wider JTE dose tolerance of 12.2 × 10^12^ cm^−2^ at 90% of the ideal BV and a positive charge density of 3.2 × 10^12^ cm^−2^ at 85% of the ideal BV. The Step-DZ-JTE with FP is introduced on the basis of a Step-DZ-JTE by adding an anode FP. The Step-DZ-JTE with FP further improves the effects of JTE dose and SC. Moreover, the Step-DZ-JTE with FP does not require an additional fabrication process. These performance improvements show that the Step-DZ-JTE with FP is a promising edge termination technique for SiC devices.

## Figures and Tables

**Table 1:** Major optimized parameters of the proposed structure.

Parameter	Value
P+ anode junction depth	0.6 μm
Junction termination extension (JTE) junction depth	0.8 μm
Depth of the step JTE (*D*_s_)	0.3 μm
Length of the step JTE (*L*_s_)	45 μm
Length of the anode FP (*W*_fp_)	20 μm
Thickness of drift	30 μm
n- drift dopant concentration (*N*_D_)	3.0 × 10^15^ cm^−3^
p^+^ anode dopant concentration (*N*_A_)	1.0 × 10^19^ cm^−3^

**Table 2:** Basic properties of different JTEs.

Structures	SZ-JTE	DZ-JTE	Step-DZ-JTE	Step-DZ-JTE with FP
JTE total length (μm)	100	100	100	100
Number of p-type implant	1	2	2	2
JTE1 dose tolerance for 90% BV (× 10^12^ cm^−2^)	0.4	4.1	12.2	13.8
Total JTE dose tolerance for 90% BV ((× 10^12^ cm^−2^)	0.4	5.6	16.3	18.4
The percentage of positive and negative variation	(+2.2%, −2.2%)	(+17.5%, −17.0%)	(+75%, −18.4%)	(+75%, −35%)
Max. positive SC density for 85% BV (× 10^12^ cm^−2^)	0.5	3.2	3.7	5.5
